# Prolonged Dyspnea after Interscalene Block: Attributed to Undiagnosed Addison's Disease and Myasthenia Gravis

**DOI:** 10.1155/2011/968181

**Published:** 2011-06-07

**Authors:** John G. Skedros, Casey J. Kiser, Shaun D. Mendenhall

**Affiliations:** Utah Bone and Joint Center, Department of Orthopaedics, Salt Lake City, UT 84107, USA

## Abstract

This report describes a patient who had a series of daily interscalene nerve blocks to treat pain following a shoulder manipulation for postsurgical stiffness. She experienced acute respiratory compromise that persisted for many weeks. All typical and unusual causes of these symptoms were ruled out. Her treating pulmonologist theorized that the ipsilateral carotid body had been injured. However, it was subsequently determined that the constellation of symptoms and their prolonged duration were best explained by a poor stress response from Addison's disease coupled with exacerbation of early onset myasthenia gravis. This patient's case is not a typical reaction to interscalene nerve blocks, and thus preoperative testing would not be recommended for myasthenia gravis or Addison's disease without underlying suspicion. We describe this report to inform physicians to consider a workup for these diagnoses if a typical workup rules out all usual causes of complications from an interscalene block.

## 1. Introduction

Interscalene brachial plexus blocks are a common adjunct for managing postoperative pain following shoulder procedures. In a study of complications resulting from interscalene brachial plexus blocks, Lenters et al. [1] compiled data from their local medical center, from three previous studies [2–4], and from the database of the American Society of Anesthesiologists (ASA). In these five data sets, there were 179 total complications in over 11,000 blocks, ranging from 0.7% (42 of 6,446 blocks; ASA) to 14.6% (76 of 521 blocks) [2]. The percentage of the total complications that involved the respiratory system ranges from zero [3] to 50% [4]. The majority of respiratory complications (80–100%) were attributed to pneumothorax, prolonged phrenic nerve palsy, and respiratory distress, usually presenting early on after the block. Less common respiratory complications include pulmonary edema and airway injury. In a study of the use of an indwelling catheter for interscalene blocks, Bryan et al. [5] reported two complications in 144 blocks (1.4%). Both complications involved the respiratory system; one was a small apical pneumothorax and the other transient shortness of breath. 

Our review of complications of interscalene nerve blocks reported in the English literature failed to locate a case report describing prolonged respiratory compromise following an interscalene block that was ultimately attributed to a poor stress response from Addison's disease in addition to influences of early onset myasthenia gravis. We report a rare case of a patient who had both of these conditions that were undiagnosed at the time she had an indwelling interscalene catheter placed. We describe the nature of the presenting symptoms in the context of the more typical complications from this procedure, and the course leading to our patient's ultimate diagnosis. While these conditions are not common, it is important that they are considered in a patient with prolonged respiratory complications after an interscalene block.

## 2. Case Report

A 40-year-old obese (height 1.7 m; weight 122.5 kg; BMI 42.3 kg/m^2^) female was scheduled for an elective manipulation of her left shoulder in April, 2003 for recurrent post-surgical stiffness, which was the consequence of a previous open subacromial decompression and distal clavicle excision performed ten months prior. This was the patient's third postoperative manipulation (previous at 2.5 and 4 months post-initial surgery) under general anesthesia for the same condition. The only routine medications taken for shoulder pain were hydrocodone bitartrate acetaminophen and diclofenac sodium. She reported no allergies to any medications or local anesthetic agents. The patient was employed as an elementary school teacher, had quit smoking cigarettes two years prior, and occasionally consumed alcoholic beverages. She had no personal history of diabetes, neuropsychiatric diagnoses, reactive airway disease, autoimmune diseases, or other pulmonary/ventilatory or cardiovascular abnormalities or conditions. A family history was not available because she was adopted as a child. A thorough review of symptoms only revealed reports of occasional fatigue.

With the patient awake and with the use of a nerve stimulator, the anesthesiologist administered a standard preoperative interscalene block [6] without the use of an indwelling catheter. The anesthesiologist routinely performed interscalene blocks with the use of a nerve stimulator (ultrasound machines were not used for interscalene blocks in our hospital at that time). A bolus injection of 25 milliliters of 0.5% levobupivacaine was administered. (Levobupivacaine is at least as potent as bupivacaine but has a superior safety profile with respect to the cardiovascular and central nervous systems [7]). Although the patient's obese neck made it difficult to discern anatomical landmarks, the block was immediately effective. General endotracheal anesthesia was then administered, and a shoulder manipulation was performed without complications. This initial interscalene block lasted approximately 14 hours, and there were no subsequent signs or symptoms of respiratory compromise or other complications. 

In the morning after the shoulder manipulation, a second interscalene block was performed and an indwelling catheter was left in place for repeated dosing [6]. The indwelling catheter was now used because it was felt that without repeated blocks the patient would have difficulty controlling her pain for daily shoulder motion exercises. This issue had not been considered at the time of the first block. 

The second block included the administration of 25 milliliters of 0.25% levobupivacaine. Although it was difficult to place the catheter due to the patient's corpulent neck, the second interscalene block was effective and no complications were recognized. The patient went home later that afternoon. On each of the next four mornings, the patient came to the hospital for a repeated bolus dose and then went home. All daily boluses provided 12–14 hours of excellent pain relief.

In the evening after the first two catheter doses, she reported “drooping” of her entire face and a brief episode of a “rapid heart rate”. After the third dose through the catheter (the second day following hospital discharge), the patient also reported new-onset shortness of breath, night sweats, and subjective fevers. In addition to bilateral facial drooping, she reported a relative increase in left eye drooping, trouble speaking, and drooling. However, the following morning (third postprocedure day) the anesthesiologist did not consider these improving signs and symptoms as concerning, and the block was redosed for the fourth time. A chest radiograph was not obtained. The motivation to continue redosing the block was to provide excellent pain control for motion exercises in a patient who was prone to developing adhesive capsulitis of the shoulder. Even in view of the aforementioned symptoms, the patient also desired to have the blocks readministered for this reason. 

On the fourth day after discharge from the hospital, the fifth, and final, anesthetic bolus was infused and the catheter removed. At home three hours later, she noted “rapid breathing” and “air hunger”. The following morning (six days after manipulation) the patient came to the emergency department with diaphoresis, dyspnea and tachypnea (20–32 breaths/minute), chest discomfort (a “heavy feeling”), and dysphonia (reduced phonation without hoarseness or dysarthria). There was no cough or sputum production, and a twelve-lead electrocardiogram (EKG) was normal. There were mild bibasilar rales and mild erythema with tenderness (but no crepitation or fluctuation) in the supraclavicular area near the catheter insertion site. Other vitals, laboratory values, and physical findings on admission and thereafter are in Table 1. The patient was found to have acute respiratory alkalosis attributed to tachypnea. A chest X-ray showed only mild basilar atelectasis and no evidence of hemidiaphragm paralysis [8]. 

Venous doppler ultrasound evaluations of the popliteal regions and left arm, and a computerized tomographic (CT) angiogram of the chest revealed no abnormality. CT scans of her neck and clavicular regions did not reveal abnormality. Inspiration and expiration chest radiographs showed normal bilateral diaphragm excursion and no evidence of hemi-diaphragm paralysis [9, 10]. Bilateral pulmonary angiograms, inferior vena cavogram (with intravenous contrast), and echocardiogram (with measurement of pulmonary arterial pressure) were within normal limits. 

There was concern that the leukocytosis (Table 1) and localized erythema could be from an infection. However, the patient was never febrile and the white blood cell count decreased without the use of antibiotics. It was hypothesized that the localized erythema was a local reaction to the anesthetic. Based on the possibility that an enterovirus was causing the pleurodynia, a throat culture was obtained and was negative. During the night, the patient's hyperventilation improved, but did not fully resolve during sleep, even after initiation of a patient-controlled analgesia (PCA) (hydromorphone) pump. Although her respiratory rate fluctuated, her heart rate was relatively consistent overnight (Table 1). 

On the second hospital day, shallow and rapid breathing had increased as high as 40 breaths/minute. Results of laboratory and other diagnostic tests ruled out pulmonary embolus, hemi-diaphragmatic paralysis, recurrent laryngeal nerve paralysis, brachial plexitis, pneumothorax, pneumonia, endocarditis, pericarditis, myocardial infarction or ischemia, and significant cutaneous or subcutaneous infection. The patient was discharged two days later on 2 L/min of oxygen with a diagnosis of dyspnea/hyperventilation of unknown cause. Two days later, PA and lateral chest radiographs showed no significant changes.

By six days after hospital discharge, the patient contacted her orthopaedic surgeon (JGS) stating that she was “short of breath all of the time”, including activities with minimal exertion such as talking. In addition, she reported having “drenching sweats” and that she would occasionally wake from sleep “short of breath”. Her respiratory rate was 20–24 breaths/minute, and there was no evidence of infection and no abnormalities on a chest radiograph. A pulmonologist performed spirometry, which showed a very mild restrictive abnormality with 78% forced vital capacity (FVC) (normal > 80%) and 80% forced expiratory volume (FEV_1_) (normal > 75%) without change after inhaled bronchodilators. Her oxygen (O_2_) saturation was 97% at rest on room air and did not change after walking 100 meters on level ground, or up and down 40 stairs. A sniff test with fluoroscopy (which detects paradoxical hemi-diaphragm motion with vigorous sniffing) showed no abnormality. The pulmonologist hypothesized that the carotid body had somehow been injured during the administration of the interscalene blocks.

Twenty-two days following hospital discharge, the patient consulted with a neurologist. Brain and neck MRIs and a left carotid neck MRA (magnetic resonance angiography) showed no abnormal findings. A specific diagnosis was not identified. At 33 days following hospital discharge, another fluoroscopic exam showed no abnormality. Arterial blood gases were consistent with mild hyperventilation. A sleep study showed no airway obstruction or sleep disorder. Her symptoms slowly improved without further intervention or workup. At six months after the final interscalene block, she reported nearly complete recovery. However, during the following year she reported having increasing mild muscle fatigue, occasional syncopal episodes, an unintentional 30 lb (13.6 kg) weight loss, poor appetite, and recurrent nausea. Medical workup eventually revealed Addison's disease, which was established by plasma cortisol measurements and a synthetic ACTH stimulation test. 

During the subsequent three years, and despite medical treatment for Addison's disease, the patient experienced worsening respiratory compromise with exertion, poor ability to maintain a conversation (dysphonia), and eventually bilateral ptosis. Further workup (five years after shoulder manipulation), including a positive serum acetylcholine receptor antibody test, revealed myasthenia gravis. In September 2010 (7 years post manipulation), the patient had a thymectomy, which successfully ameliorated the majority of her residual symptoms.

## 3. Discussion

What is clear in this complex case is that the patient's prolonged respiratory distress could not, at that time, be explained in a simple anatomical or physiological context despite a comprehensive workup investigating the typical causes of respiratory complications following an interscalene nerve block. These symptoms became recognizable later when additional workup revealed the rare coexistence of Addison's disease with myasthenia gravis. 

Addison's disease is rare, with a prevalence of 4 to 11 out of 100,000 [11]. Typically, patients with chronic adrenal insufficiency that characterizes Addison's disease and who undergo surgery are given perioperative injections of intravenous corticosteroids in order to avoid precipitating adrenal crisis [12]. Addison's symptoms usually progress slowly and typically are not recognized as being important until a stressful event like illness, surgery, or an accident occurs—such events account for 25% of cases when the first symptoms are recognized [11]. Significantly low levels of cortisol likely caused our patient to respond poorly to the acute physiological stress of the shoulder manipulation and subsequent series of interscalene blocks. This interpretation is consistent with reports showing that Addison's disease can present with compromised pulmonary symptoms due to respiratory muscle weakness [13, 14].

Myasthenia gravis is also associated with poor respiratory reserve, which may have contributed to our patient's prolonged symptoms [15]. Myasthenia gravis is an autoimmune disease characterized by weakness and fatigability of skeletal muscles, with improvement following rest [16]. In contrast to Addison's disease, perioperative use of corticosteroids is uncommon in patients with stable myasthenia gravis who have elective surgery [17]. 

The association of myasthenia gravis with Addison's disease is very rare but has been reported in the literature [18, 19], although not typically presenting in the perioperative setting. Nearly four years following the interscalene blocks, our patient began noticing significant muscle weakness and then later ptosis. This later occurrence of ptosis makes her presentation unusual because in a large majority of cases ptosis usually precedes muscle weakness [20–22]. Similar to our patient, myasthenia gravis symptoms usually present with fluctuating, asymmetrical stress-dependent weakness in the absence of other neurological disturbances [23]. Muscle weakness in association with myasthenia gravis could have produced a feeling of dyspnea. However, a more convincing case for attributing her symptoms to myasthenia gravis would have been the occurrence of hypercarbic respiratory acidosis (which was not seen) along with direct measurement of respiratory muscle strength. Consequently, we conclude that most of her acute symptoms were best attributed to an Addisonian crisis. 

The explanation that the carotid body and possibly nearby cranial nerves were somehow indirectly or directly affected by the blocks (e.g., direct injury from the needle, wayward catheter placement, and/or local adverse effect of the anesthetic) did not explain her *bilateral* facial drooping. In retrospect, this “injury/irritation interpretation” was not tenable in view of (1) pulmonary complications from local anesthesia for carotid endarterectomy have not been reported in a large number of studies [24], and (2) carotid body abnormalities might affect respiratory rate but not other pulmonary function tests (our patient had involvement in both). For example, in the past when the carotid body was surgically removed to increase arterial oxygen saturation as a treatment for chronic obstructive pulmonary diseases, increased diaphragm excursion occurred with little change in respiratory rate [25, 26]. Additionally, injury to the carotid body would have been expected to relieve dyspnea (at least partially) by reducing the hypoxic and CO_2_ ventilatory drive [27].

Unfortunately the patient's reports of bilateral facial drooping, dysphonia, and drooling, had nearly resolved by the time the first four blocks were redosed through the catheter. Consequently, her description was not only questioned but also led to a focus on her unilateral facial signs and on the initial speculation that she had features of Horner's syndrome, which is common after interscalene nerve blocks [28–31]. If our patient actually had only transient Horner's syndrome, then only ptosis of the ipsilateral superior tarsus (eyelid) and miosis (constriction) of the ipsilateral pupil would have occurred—not entire facial drooping. 

In conclusion, the acute respiratory compromise and the additional symptoms that occurred in our patient following a series of interscalene blocks can be best explained by a poor stress response from Addison's disease coupled with exacerbation of early onset myasthenia gravis. Although we did not obtain definitive data confirming the dual diagnoses at the time of her symptoms, this is the most complete explanation available. This patient's case is not a typical reaction to an interscalene nerve block, and thus preoperative testing would not be recommended for myasthenia gravis or Addison's disease without underlying suspicion.

## Figures and Tables

**Table 1:** Second hospitalization (6 days after shoulder manipulation).

	Day 1 of 2nd hospitalization	Night 1 of 2nd hospitalization	Day 2 of 2nd hospitalization	Day 4 of 2nd hospitalization
Respiratory rate	20–32 breaths/minute	13–40 breaths/minute	40 breaths/minute	
Oxygen saturation	97% on 3 liters of oxygen 88% on room air		92–96% on 2 liters of oxygen	93% on room air
Heart rate	66 beats/minute	65–75 beats/minute	85–95 beats/minute	
Blood pressure	135/95		130/80	
White blood cell count (normal range 4,500–10,000 cells/*μ*L)	18,000 cells/*μ*L	16,200 cells/*μ*L	11,600 cells/*μ*L	10,400 cells/*μ*L
Temperature	Afebrile	Afebrile	Afebrile	Afebrile
Hematocrit, hemoglobin, red blood cell count, platelets	All Within Normal Limits			
Comprehensive metabolic panel (glucose, calcium, albumin, total protein, sodium, potassium, chloride, BUN, creatinine, AST, ALT, ALP, bilirubin)	All Within Normal Limits			
Creatine kinase (CK) (normal range 96–140 units/L)	330 units/L			147 units/L
CKMB (normal range 0.0–5.0 ng/mL)	0.9 ng/mL			1.3 ng/mL
CK index (normal range 0.3–0.9%)	0.30%			0.90%
Troponin (normal range 0.3–2.0 ng/mL)	0.3 ng/mL			
D-Dimer (normal range 68–494 ng/mL)	1256 ng/mL			
Fibrinogen (normal range 148–413 mg/dL)	499 mg/dL			
pH (normal 7.35–7.45)	7.58			
pCO_2_ (normal range 35–45 mmHg)	23 mmHg			
pO_2_ (normal range 80–100 mmHg)	85 mmHg			
Bicarbonate (normal range 19–25 mEq/L)	20.8 mEq/L			
Base excess (normal range −2.5–2.5 mEq/L)	1.1 mEq/L			
Lactic acid (normal range 0.7–2.1 mmol/L)	2.0 mmol/L			
Erythrocyte sedimentation rate (normal range 0–20)	36			
